# Non-Diethylstilbestrol-Associated Primary Clear Cell Carcinoma of the Vagina: Two Case Reports with Immunohistochemical Studies and Literature Review

**Published:** 2014-05

**Authors:** Shagufta T. Mufti, Hiba Hassan Ali

**Affiliations:** Department of Anatomic Pathology, Faculty of Medicine, King Abdulaziz University, Jeddah, Saudi Arabia

**Keywords:** Diethylstilbestrol, Clear cell carcinoma, Vagina

## Abstract

Primary clear cell adenocarcinomas most commonly involve the genitourinary system, including the vagina. Previously, primary clear cell adenocarcinomas of the vagina have been discussed within the context of prenatal exposure to diethylstilbestrol. Due to its widely proven role in the development of this carcinoma, administration of diethylstilbestrol is prohibited. We present two cases of non-diethylstilbestrol-associated primary clear cell adenocarcinoma of the vagina from the archives of the Anatomical Pathology Department at King Abdulaziz University in order to improve our understanding of its biological behavior. Our findings suggest that primary clear cell adenocarcinoma of the vagina may be unrelated to diethylstilbestrol exposure and that non-diethylstilbestrol-associated primary clear cell adenocarcinoma of the vagina, when present at a younger age, may have a worse prognosis.

## Introduction

Primary clear cell adenocarcinomas (PCCA) most commonly involve the vagina, cervix, ovaries, and urinary tract including the kidneys. These carcinomas are histologically^1^ and immunohistochemically^2^ identical. Primary clear cell carcinoma of the vagina (PCCAV) accounts for 5%-10% of all vaginal cancers.^1^ Over the last 35 years, PCCAV has been discussed within the context of prenatal exposure to diethylstilbestrol (DES).^3^ From 1938 until 1971, DES was used to prevent miscarriages.^4^ In view of its proven role in the development of PCCAV, DES was withdrawn from wordwide use by the FDA.^4^ However, on occasion, cases of PCCAV have been reported in the literature although its diagnosis is markedly less common compared to the 1980s^5^ with most cases reporting no history of DES exposure.^6^ In Saudi Arabia, most vaginal cancers are squamous cell carcinomas (90%).^7^ To the best of our knowledge no cases of non-DES-associated PCCAV have previously been reported to the literature from Saudi Arabia. Little is known about the nature of PCCAV that occurs in the absence of DES exposure. Information on the clinical behavior, pathology and prognosis of non-DES-associated PCCAV is sparse and inconsistent as they are rare. The purpose of this study was to report two cases of non-DES-associated PCCAV in our effort to further improve the understanding of the biological behavior of these rare tumors in terms of prognosis.

## Case 1

**Figure 1:**
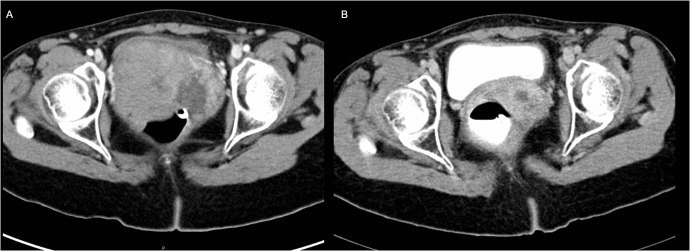
Radiographic images of Case 1. Pelvic computerized tomography (CT) scan: (A) Left image at time of presentation that shows a large hetergenous pelvic mass (9×8.8×5.5 cm) involving the vagina and upper part of the cervix. (B) Right image is taken post-radiotherapy and shows significant interval regression of the mass size.

**Figure 2:**
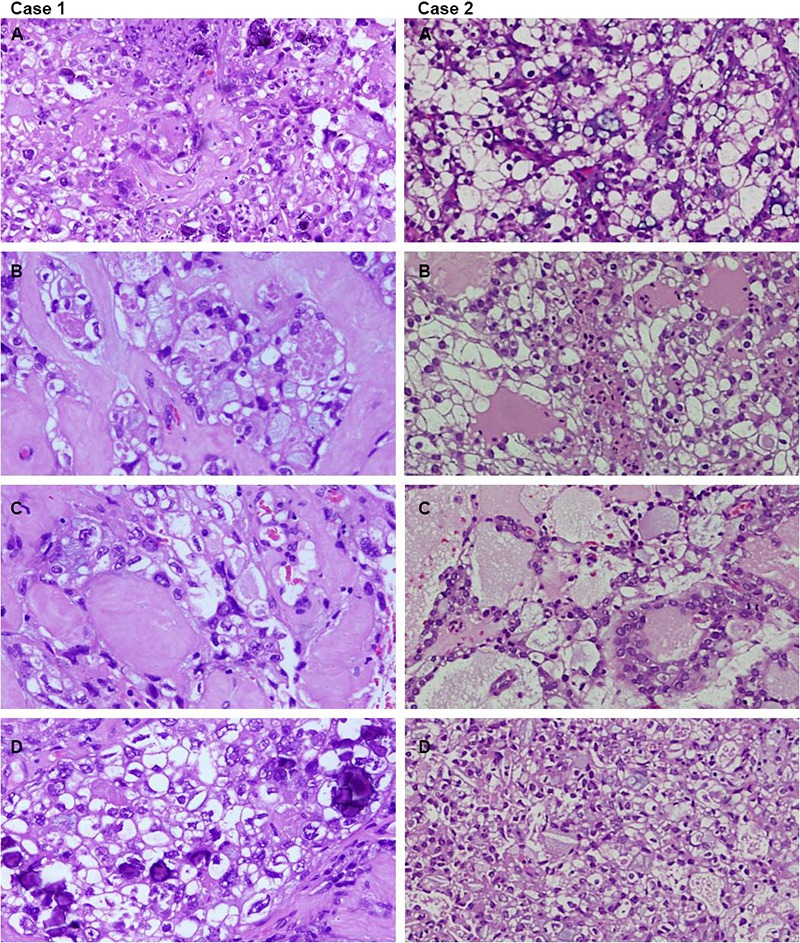
Microscopic features of both cases (hematoxylin and eosin, 400×). Left column (Case 1): Large polyhedral malignant cells with sharply demarcated cell membranes, ample clear to granular cytoplasm and pleomorphic hyperchromatic nuclei with prominent nucleoli. Occasional bizarre cells and mitosis are also seen (A & B: Upper and lower left). Sclerotic papillae with occasional bizarre cells and calcifications post-radiotherapy (C & D: Upper-middle and lower-middle left). Right column (Case 2): Large clear cells having high nuclear to cytoplasmic ratio, hyperchromasia, irregular nuclear membranes (A & B: Upper and lower right).Neoplastic growth with focal glandular, tubulocystic and pseudopapillary patterns (C & D: Upper-middle and lower-middle right).

A 27-year-old single Yemeni woman with no history of illness or prenatal DES exposure presented to the gynecology clinic with abnormal vaginal bleeding for one month duration. Although the patient’s mother was born in 1952 which was during the DES era, she had all previous normal and spontaneous term deliveries (para 6) with no history of miscarriages. Computerized tomography (CT) scan (figure 1A) and magnetic resonance imaging (MRI) revealed a large encapsulated mass that measured 9×8.8×5.5 cm located in the upper anterior vaginal wall and filled the enlarged vagina up to the left upper part of the cervix. The uterus, fallopian tubes, ovaries, rectum and urinary bladder were free from involvement. Minimal ascites and multiple enlarged external and internal left iliac lymph nodes were identified. The partially excised mass grossly measured 9×7.5×3 cm and was polyploid, grey white, necrotic and hemorrhagic. Histopathological examination revealed a neoplastic growth composed of pseudopapillary and a tubular pattern with large polyhedral malignant cells that had sharply demarcated cell membranes, ample clear to granular cytoplasm and pleomorphic hyperchromatic nuclei with prominent nucleoli and occasional mitoses (figures 2A, B case 1). Extensive areas of hemorrhage and necrosis were present with occasional bizarre cells. The mass infiltrated the vaginal wall with extension to the cervix. There was no evidence of vaginal adenosis.

**Figure 3:**
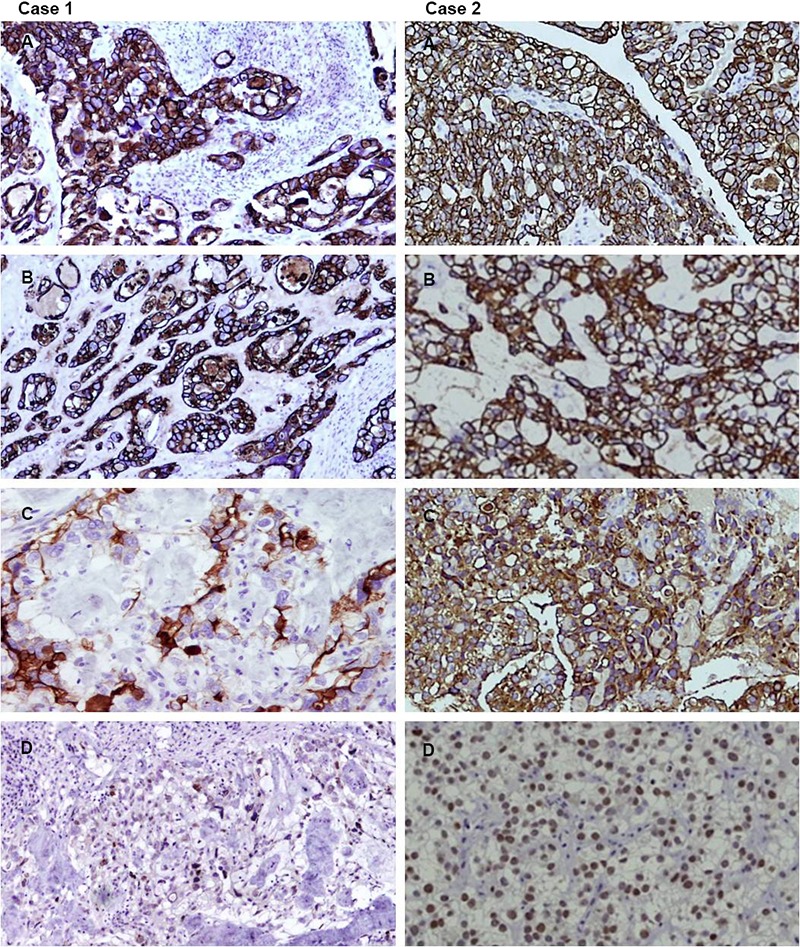
Immunohistochemical (IHC) evaluation of both cases (200×). Left column (Case 1): Tumor cells are diffusely strongly positive for CKPAN (A: upper left) and CK7 (B: upper-middle left). Tumor cells show focal positivity for CA-125 (C: lower-middle left) and p53 (D: lower left). Right column (Case 2): Tumor cells diffusely and strongly express CKPAN (A: upper right), CK7 (B: upper-middle right), CA-125 (C: lower-middle right) and p53 (D: lower right).

The tumor cells were diffusely and strongly positive for CKPAN (figure 3A case 1) and CK7 (figure 3B case 1). In addition, they were focally positive for CA-125 (figure 3C case 1) and p53 (figure 3D case 1), with weak, focal expression of CEA, EMA, bcl-2, and CD15. The cells were negative for CK20, β-hCG, renal cell carcinoma (RCC) antibody, alpha 1-fetoprotein and CD 30.

The patient received radiotherapy and on follow-up significant regression in the tumor bulk was apparent radiologically (figure 1B) and to a lesser degree in the enlarged lymph nodes. The remaining tumor was excised three months later. Microscopic evaluation revealed the same findings with sclerotic papillae and frequent calcifications (figures 2C, D case 1). Two months later the patient underwent total abdominal hysterectomy. Grossly there were no findings suggestive of prenatal DES exposure such as cervical hypoplasia, pseudopolyp, or coxcomb deformity. Microscopically, the remaining vagina and cervix were negative for tumor cells. The patient was classified as stage III. Radiological and pathologic examinations revealed that the tumor was confined to the vaginal wall (T_1_); lymph node metastasis was diagnosed radiologically (N_1_); and there was no distant metastasis identified, neither clinically or radiologically (M_0_). At two years follow-up the patient remains well with no evidence of recurrence.

## Case 2

**Figure 4:**
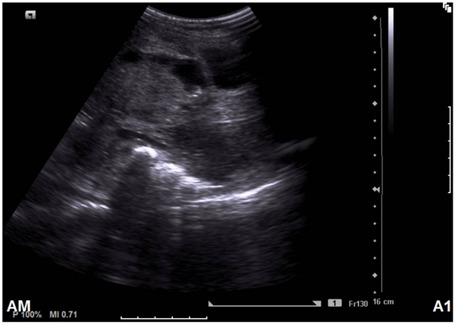
Radiographic image of Case 2. Pelvic ultrasound  shows hypoechoic predominantly endoluminal hetergenous  mass measuring 5×4.5×4.8 cm distending the upper vagina.

A 9-year-old Ethiopian girl with no history of prenatal DES exposure presented to the gynecology clinic with abnormal vaginal bleeding. The patient’s mother was born in 1973, three years later than the period considered as the DES era. She was para 4 with all normal spontaneous term deliveries and no history of miscarriages. On chest and abdominal examination the patient had bilateral pleural effusion, hepatomegaly and ascites. CT and ultrasound (figure 4) revealed a heterogeneous mass that measured 5×4.8×4.5 cm located in the anterior vaginal wall. Radiologically, the uterus, cervix, fallopian tubes ovaries, rectum and urinary bladder were free of tumor involvement. Massive ascites and multiple liver secondaries were also identified on CT scan. Pelvic examination performed under anesthesia revealed a fungating, polypoid mass arising in the upper third of the anterior vaginal wall. No abnormality was detected in the uterus, cervix or ovary intra-operatively. The mass was surgically excised with a gross measurement of 3.5×2×0.5 cm and was polypoid, grey-white, necrotic and hemorrhagic. Histopathological examination revealed a polypoid neoplastic growth with focal glandular, tubulocystic and pseudopapillary patterns (figures 2 C, D case 2) composed of large clear cells that had high nuclear-to-cytoplasmic ratio, hyperchromasia, irregular nuclear membranes and frequent mitoses (figures 2A, B case 2). Frequent hobnail cells were seen. The background was necrotic and hemorrhagic. The mass was superficial with minimal infiltration of the vaginal wall with no evidence of vaginal adenosis. The tumor cells diffusely and strongly expressed CKPAN (figure 3A case 2), CK7 (figure 3B case 2), CA-125 (figure 3C case 2), and p53 (figure 3D case 2). They were also positive for bcl-2, CEA and focally positive for EMA and CD15 but negative for CK20, RCC antibody, β-hCG, alpha 1-fetoprotein and CD 30.The patient was considered as stage IVB. Radiological and pathological examination revealed that the tumor was confined to the vaginal wall (T_1_) with no regional lymph node metastasis identified (N_0_). Distant metastasis to the liver was radiologically diagnosed (M_1_). The patient had a short course of treatment and died postoperatively.

## Discussion

Recent studies suggest that non-DES-associated and DES-associated PCCAV have different natural histories.^6^ The literature lacks information regarding the status of current or past prescription practices of DES in the Far East, Middle East and Africa, including countries such as Saudi Arabia, Yemen and Ethiopia. This lack of information has further limited our knowledge regarding its carcinogenic role in these regions. However according to the National Drug and Poison Information Center of the Saudi Food and Drug Authority, after a reported relationship of parenatally administered DES to adenocarcinoma, the use of DES during pregnancy was banned in the 1980s.^8^ There was only one case of DES-associated PCCAV reported in Saudi Arabia.^9^ Both patients in our study had no histories of DES exposure which was additionally supported by the uneventful, normal obstetric histories of their mothers. Specifically there was no history of miscarriages or premature births which excluded any DES-induced influence. There was also no clinical evidence suggestive of other primary tumors to consider metastasis.

A study of 28 cases^6^ and a few case reports of non-DES-associated PCCA of vagina^1^^,^^6^^,^^10^^-^^12^and cervix^13^^,^^14^ have been reported over the past decade. Although DES has reportedly not been used as treatment for threatened abortion in Japan, at least nine cases of PCCA of the vagina and cervix have been reported over the past two decades.^11^ Abnormal vaginal bleeding, discharge, dyspareunia and vaginal mass are the most common presentations.^1^^,^^6^ Non DES PCCV shows a bimodal age distribution with the first peak observed at 26 years and the second at 71 years of age.^1^^,^^6^ A different subset of patients with non-DES-associated PCCAV in postmenopausal women and prepubertal girls has also been reported^13^ with a grave prognosis.^6^

Gross tumor size varies from microscopic to 10 cm and is described as either a polypoid, nodular, flat or ulcerated mass. Microscopically this tumor show a predominantly tubulocystic pattern followed by solid and papillary patterns. However, a mixture of types is common. These structures are lined by cuboidal, hobnail or flat cells. Cytoplasmic clearing is due to the presence of glycogen. Cords having eosinophilic cytoplasm may also be present. Nuclear pleomorphism is variable with mitosis usually less than 10/10 high power fields. The characteristic immunoprofile of PCCA of the genitourinary tract for all sites is CK7, CAM5.2, 34 beta E12, CEA, C-A125, Leu-M1 and vimentin positive.^2^ They over express p53 and bcl-2 and exhibit variable positivity for estrogen and progesterone receptors and HER2 neu. Both cases have shown IHC positivity to CK-PAN, CK7, CA-125 and p53. They also focally expressed CEA, EMA, bcl-2, and CD15 but were negative for myogenin, desmin, vimentin and RCC antigen. PCCA tumor cells are negative for CK20, β-hCG and alpha 1-fetoprotein.^2^ These markers assist with the differentiation of PCCAV from other tumors in this location such as yolk sac tumor, sarcoma botryoides, embryonal carcinoma, and metastatic RCC.

Non-DES-associated PCCA of the vagina and cervix may also be related to adenosis and other congenital malformations such as didelphys uterus with a double vagina, renal agenesis and situs inversus.^6^^,^^11^^,^^12^ Although adenosis is detected around the PCCAV and believed to be the precursor of PCCA there is no sound scientific evidence that confirms a causal or interdependent relationship between adenosis, PCCAV and DES exposure. Ongoing publication of non-DES associated PCCAV data in the literature, particularly from countries like Japan that have not prescribed DES raises the possibility that adenosis may be a step in the pathogenesis of PCCAV regardless of presence or absence of DES exposure. At the molecular level Watanabe et al.^10^ have suggested that stability of the microsatellite loci and overexpression of p53 protein without p53 gene mutation is a biologic cellular characteristic of non-DES-associated sporadic PCCAV.

Non-DES-associated PCCAV has a poor prognosis and significantly worse outcomes than those seen in patients with other primary carcinomas of the vagina. Local and distant recurrence rates are also more common among these patients than patients with squamous cell carcinoma who have received similar treatment.^6^

## Conclusion

Both cases of non-DES-associated PCCAV in our study shared common histopathological and immunohistochemical (IHC) features although they varied in their clinical outcomes. Our findings have suggested that PCCAV can be unrelated to DES exposure and this exposure may be one of the many other less known initiators of PCCAV carcinogenesis. Non-DES associated PCCAV in the pediatric age group possibly has a worse prognosis which suggests that age may be a parameter to predict biological behaviour. 

Continued monitoring of the cancer experience of the present population will be required to understand the pathogenesis of PCCAV in the absence of prenatal DES exposure and in cases that differ from PCCAV following DES exposure. This will place therapeutic implications in a different perspective for these two categories. Limitations to ascertain the third-generation carryover effects of in utero DES exposure, however, also remain a possibility to be considered.
